# The adipokinetic hormones and their cognate receptor from the desert locust, *Schistocerca gregaria*: solution structure of endogenous peptides and models of their binding to the receptor

**DOI:** 10.7717/peerj.7514

**Published:** 2019-08-30

**Authors:** Graham E. Jackson, Elumalai Pavadai, Gerd Gäde, Niels H. Andersen

**Affiliations:** 1 Department of Chemistry, University of Cape Town, Cape Town, Western Cape, South Africa; 2 Department of Physiology and Biophysics, Boston University, Boston, MA, USA; 3 Department of Biological Sciences, University of Cape Town, Cape Town, Western Cape, South Africa; 4 Department of Chemistry, University of Washington, Seattle, WA, USA

**Keywords:** Adipokinetic hormones, *Schistocerca gregaria*, Locmi-AKH-I, Aedae-AKH, Schgr-AKH-II, *Locusta migratoria*, Homology modeling, Agonist docking

## Abstract

**Background:**

Neuropeptides exert their activity through binding to G protein-coupled receptors (GPCRs). GPCRs are well-known drug targets in the pharmaceutical industry and are currently discussed as targets to control pest insects. Here, we investigate the neuropeptide adipokinetic hormone (AKH) system of the desert locust *Schistocerca gregaria*. The desert locust is known for its high reproduction, and for forming devastating swarms consisting of billions of individual insects. It is also known that *S. gregaria* produces three different AKHs as ligands but has only one AKH receptor (AKHR). The AKH system is known to be essential for metabolic regulation, which is necessary for reproduction and flight activity.

**Methods:**

Nuclear magnetic resonance techniques (NMR) in a dodecylphosphocholin (DPC) micelle solution were used to determine the structure of the three AKHs. The primary sequence of the *S. gregaria* AKHR was used to construct a 3D molecular model. Next, the three AKHs were individually docked to the receptor, and dynamic simulation of the whole ligand–receptor complex in a model membrane was performed.

**Results:**

Although the three endogenous AKHs of *S. gregaria* have quite different amino acids sequences and chain length (two octa- and one decapeptide), NMR experiments assigned a turn structure in DPC micelle solution for all. The GPCR-ModSim program identified human kappa opioid receptor to be the best template after which the *S. gregaria* AKHR was modeled. All three AKHs were found to have the same binding site on this receptor, interact with similar residues of the receptor and have comparable binding constants. Molecular switches were also identified; the movement of the receptor could be visually shown when ligands (AKHs) were docked and the receptor was activated.

**Conclusions:**

The study proposes a model of binding of the three endogenous ligands to the one existing AKHR in the desert locust and paves the way to use such a model for the design of peptide analogs and finally, peptide mimetics, in the search for novel species-specific insecticides based on receptor–ligand interaction.

## Introduction

In 1976 the primary structure of the first metabolic insect neuropeptide was published (Stone et al., 1976). The decapeptide was isolated from the retrocerebral glands (corpora cardiaca) of the migratory (*Locusta migratoria*) and desert (*Schistocerca gregaria*) locusts. This neuropeptide was functionally paramount in mobilizing lipids, especially during flight episodes and, hence, was classified as an adipokinetic hormone (AKH). Its modern code name is Locmi-AKH-I (for primary structure, see Table 1). Subsequently, in both locust species, a second species-specific AKH octapeptide was found (Gäde et al., 1986; Siegert, Morgan & Mordue, 1985) (see Table 1). Later, a third AKH, again an octapeptide, was isolated and functionally characterized from *L. migratoria* (Oudejans et al., 1991) (see Table 1). Genome data mining lead to the discovery of a putative fourth AKH in the migratory locust (Veenstra, 2014). The sequence of this octapeptide was identical to an AKH, Aedae-AKH, previously cloned from the yellow fever mosquito, *Aedes aegypti* (Kaufmann, Merzendorfer & Gäde, 2009). It was also found in the corpora cardiaca of the alderfly, *Sialis lutaria* (Gäde, Šimek & Marco, 2009) and the desert locust (Marchal et al., 2018).

**Table 1:** Primary structure of the adipokinetic peptides of locusts.

Peptide name	Sequence	Species
Locmi-AKH-I	p**EL**N**F**TPN**W**GT amide	*L. migratoria*, *S. gregaria*
Locmi-AKH-II	p**EL**N**F**SAG**W** amide	*L. migratoria*
Locmi-AKH-III	p**EL**N**F**TPW**W** amide	*L. migratoria*
Locmi-AKH-IV (= Aedae-AKH)	p**EL**T**F**TPS**W** amide	*L. migratoria*, *S. gregaria*
Schgr-AKH-II	p**EL**N**F**STG**W** amide	*S. gregaria*

**Note:**

Conserved residues are highlighted in bold and underlined.

All these peptides are members of the large AKH/red pigment-concentrating hormone (RPCH) family, which occurs not only in insects and crustaceans, but also evolved in molluscs (Johnson et al., 2014; Li et al., 2016). The AKH gene codes an mRNA that is translated into a pre-propeptide with the following features: a signal peptide is followed immediately by the respective AKH peptide, a glycine amidation site, a dibasic processing site and, C-terminally, another putative peptide of variable length (Gäde & Marco, 2013). After cleavage and post-translational modification, the structure of the mature AKH is characterized by a chain length of 8–10 amino acids, a pyroglutamate residue at the N-terminus and a carboxyamide at the C-terminus. The amino acids Leu, Ile, Val, Tyr, or Phe are found at position 2, Asn or Thr at position 3, the aromatic residues Phe or Tyr at position 4, Ser or Thr at position 5, and various amino acids at position 6, 7, and 10. The aromatic Trp is always present at position 8 and Gly at position 9 (Gäde, 2004, 2009).

Most insect neuropeptides, including AKHs, exert their activity via binding to G protein-coupled receptors (GPCRs). In the pharmaceutical industry GPCRs are well-known drug targets, and insect neuropeptide GPCRs are being considered as targets for the control of pest insects (Audsley & Down, 2015; Verlinden et al., 2014). A number of AKH receptors (AKHRs) have been cloned and sequenced (Alves-Bezerra et al., 2016; Caers et al., 2012; Hou et al., 2017, to name a few) and the AKH system with its primarily metabolic function has been identified as a putative target as well (Gäde & Goldsworthy, 2003). With this aim, recent studies (Marchal et al., 2018) characterized the AKHR from the desert locust and used a mammalian cell-based bioluminescence assay to investigate activation of the cloned receptor by a number of naturally-occurring peptides (Marchal et al., 2018).

The first 3D-structure of an AKH hormone peptide derived from nuclear magnetic resonance (NMR) spectroscopy was published by Zubrzycki & Gäde (1994). Modeling of binding of members of the AKH/RPCH family to their cognate receptors has also been undertaken before: one model has been proposed for an insect, the malaria mosquito, *Anopheles gambiae* (Mugumbate et al., 2013) and another for a crustacean, the water flea *Daphnia pulex* (Jackson et al., 2018). Although the receptors share spatial regions, the binding modes of the two ligands have different orientation (Jackson et al., 2018). In both cases, however, the receptor has only one endogenous, octapeptide ligand. In the current study, three AKH neuropeptides (one decapeptide and two octapeptides, see Table 1) are present in *S. gregaria*; two of these peptides have been found to be active in lipid mobilization (Gäde, 1990) and two of these peptides bind to the same receptor (Marchal et al., 2018). The challenge, hence, was to understand how such different peptides could all activate the same receptor. The available information on the peptides and the AKHR of *S. gregaria*, gives us the opportunity to model the binding of these three peptides to their cognate receptor. This information could then be used to explain how the different peptides are able to activate the same receptor. This step is necessary in order to design novel peptidomimetic agonist or antagonists, which can be synthesize and tested as cheap pest insect control substances.

## Materials and Methods

Schgr-AKH-II was synthesized by GL Biochem Ltd. (Shanghai, China), Aedae-AKH by Pepmic Co., Ltd. (Suzhou, China), and Locmi-AKH-I by Peninsula Laboratories (Belmont, CA, USA). Purity was checked with HPLC-MS and it was found to be >95–98% pure. The peptides were not sufficiently soluble in water for the NMR experiments and so solutions were prepared in 30% dimethylsulfoxide (DMSO) and/or in a dodecylphosphocholin (DPC) micelle solution. Typically, one mg of sample was dissolved in 0.5 ml of either 20 mM phosphate buffer + 30% DMSO or 10:1 (v/v) H_2_O:D_2_O solution which was 150 mM in deuterated DPC-d38 (Cambridge Isotopes, 98.6% d) and buffered with 20 mM potassium phosphate buffer. Peptide–peptide interactions were minimized by maintaining a peptide to micelle ratio of 1:3, assuming 50 molecules of DPC per micelle (Jackson et al., 2018). Sodium 4,4-dimethyl-4-silapentane-1-sulfonate was used as an internal standard.

Nuclear magnetic resonance experiments were performed on either, a Bruker Advance 700 MHz spectrometer or a Bruker Advance 600 MHz spectrometer with a prodigy probe. A mixing time of 60 ms was used for total correlation spectroscopy (Hwang & Shaka, 1995). A mixing time of 150 ms was used for nuclear Overhauser spectroscopy (Braunschweiler & Ernst, 1983). Spectral assignments were based on the method of (Wüthrich, 1986; Weber, Morrison & Hare, 1988). ^13^C assignments were based on heteronuclear single quantum coherence spectra (Sklenar et al., 1993).

### Peptide molecular dynamics

The three AKH peptides were built using Maestro (Schrödinger, Inc., New York, NY, USA) and energy minimized using a steepest descent algorithm. NMR restrained molecular dynamic (MD) simulations in vacuum, water and DPC were performed using GROMACS version 5.1.2 (Van Der Spoel et al., 2005). Although only the final results in DPC micelle solution are relevant to the current study, the three different environments were used to adequately search conformational space in a reasonable amount of time. Thus, the vacuum simulation was done at 600 K to allow the peptide to move faster and overcome any conformational barriers. All simulations were performed using the OPLS-AA all-atom force field with a time step of two fs. The LINCS algorithm was used to constraint all bonds. A cut-off of 1.0 nm was used for van der Waals interactions and electrostatic interactions for real space calculations. Vacuum simulations were first done to search conformational space by collecting 100 snapshots of the trajectory during a 10 ns simulation, at 600 K. Each conformation was then annealed to 300 K over 40 ps. Cluster analysis of the resulting structures, using the linkage algorithm of GROMACS and a cut-off of 0.1 nm on the backbone atoms, gave one single large cluster. The conformer in the cluster with the lowest energy was used for simulations in water. Using the tip4p water model, a box containing the peptide and ~7,000 water molecules was constructed. Following equilibration, MD was performed for 10 ns at 300 K under NPT conditions. In total, 200 structures were collected at 50 ps intervals. Cluster analysis was performed as before and the results used in the DPC/water simulations.

For simulations in a water/DPC mixture, the lowest energy structure obtained previously was placed in the center of a seven nm cubic box filled with ~10,000 SPC water molecules and a 52 DPC molecule micelle as obtained from Tieleman, Van Der Spoel & Berendsen (2000). The micelle was translated so that the center of the micelle was at the bottom edge of the box. This meant that, using periodic boundary conditions, half the micelle was at the bottom of the box and the other half was at the top. The peptide was then placed in the center of the box. Energy minimization was carried out using the steepest descent method to a tolerance of 10 kJ/mol or to machine precision. Two stages of system equilibration were performed to solvate the peptide and to achieve a steady state starting temperature, pressure, and density. The first stage of equilibration involved performing MD for 100 ps under NVT conditions at 300 K followed in the second stage by a further 1 ns MD under NPT conditions. The final MD simulation was for 10 ns during which 200 snapshots were collected. Cluster analysis was performed in the same manner as before.

### Homology modeling

The primary sequence of the AKH receptor from *S. gregaria*, Schgr-AKHR, was obtained from the GenBank (GenBank ID: AVG47955.1). Transmembrane (TM) helix predictions were computed online using PSIPRED (http://bioinf.cs.ucl.ac.uk/psipred/) (Jones, 2007; Jones, Taylor & Thornton, 1994; Nugent & Jones, 2009). The results showed that this sequence had seven TM helices (Fig. S1).

The GPCR-ModSim Web server (http://gpcr-modsim.org/) (Rodríguez, Bello & Gutiérrez-De-Terán, 2012) was used for template selection and preliminary sequence alignment of Schgr-AKHR. This server accepts an amino acid sequence as input and searches templates by multiple sequence alignments with the query sequence. GPCR-ModSim identified the human kappa opioid receptor (hĸ-OR) (PDB ID: 4DJH; Wu et al., 2012) as the best template. Subsequently, the sequence alignment between Schgr-AKHR and hĸ-OR was manually edited to remove gaps in the TM domains without disrupting their conserved regions. Finally, homology models for Schgr-AKHR were constructed using Modeler 9v7. This is an automated homology modeling program that performs automated protein homology modeling and loop modeling for the receptor by satisfaction of spatial restraints (Sali, 1995). The quality of the selected model was evaluated for internal consistency and reliability, such as stereochemical quality, using PROCHECK (Laskowski et al., 1996). The quality of the non-bonded atom interactions was evaluated using ERRAT (Colovos & Yeates, 1993).

### Docking studies

The validated Schgr-AKHR model and the structure of the DPC micellar structure of the three AKH peptides were prepared for docking simulations using *Protein Preparation Wizard* and *LigPrep* of the Schrödinger suite (Schrödinger Inc., New York, NY, USA). Site-directed mutagenesis studies (Kooistra et al., 2013), molecular modeling and structural analyses (Li, Hou & Goddard, 2010) suggest that most of the class A GPCRs share a similar binding pocket *c.f*. retinal bound to rhodopsin, carazolol bound to beta-2 adrenergic receptor (Vilar et al., 2011) and Anoga-HrTH bound to the AKHR of *Anopheles gambiae* (Mugumbate, Jackson & Van Der Spoel, 2011). Thus, the extracellular portion of the receptor was used for the docking simulations.

*GLIDE docking* (Trott & Olson, 2010) was used for peptide docking with a grid space of 72 × 72 × 72, which covered all extracellular loops and helices. The receptor grid was generated for peptide ligands and the docking precision was SP-Peptides. This setting automatically increases the number of poses collected.

### MD of docked structure

The best poses from the docking studies were used as starting structures for a 2 μs MD simulation in a 1-palmitoyl-2-oleoyl-glycero-3-phosphocholine (POPC) membrane. Using the CHARMM-GUI (http://www.charmm-gui.org/) the docked complex was placed in a POPC membrane (128 POPC molecules) such that it spanned the membrane. The construct was then converted to an OPLS-AA, all–atom, force field. Using GROMACS, ~12,000 water molecules were added and the charge neutralized by adding 19 Cl^−^ ions. Several steps of equilibration were used, to pack the membrane around the receptor. This was followed by 1 μs of NPT simulation at 300 K with Berendsen pressure coupling (Berendsen et al., 1984) and a tau-p of 2.0. The free energy of binding of the final structures, from the dynamic simulations, was calculated using Prime-MM-GBSA (Schrödinger Inc., New York, NY, USA).

## Results

### Spectral assignment

**Figure 1:**
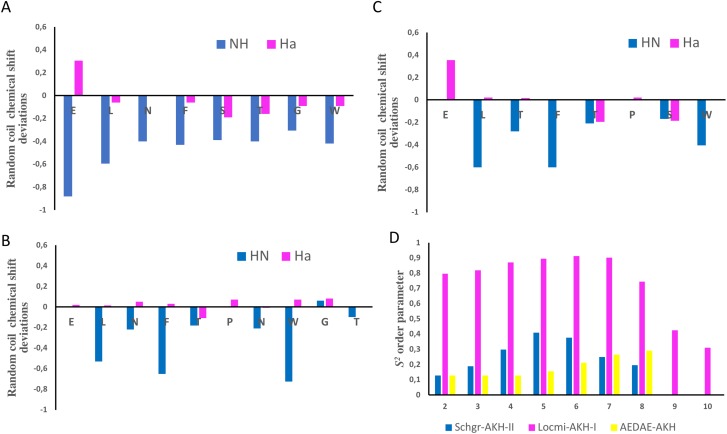
NMR chemical shift deviations and model-free order parameters of Schgr-AKH-II, Locmi-AKH-I, and Aedae-AKH in DPC micelle solution. Plots of H_α_ and H^N^ random coil chemical shift deviations of: (A) Schgr-AKH-II, (B) Locmi-AKH-I, (C) Aedae-AKH, and (D) model-free order parameter, *S*^2^.

The chemical shift assignments of the three AKH ligands in DPC are given in Tables 2–4. Previously, Berjanskii & Wishart (2006) as well as Tremblay, Banks & Rainey (2010) have shown that the chemical shifts of peptides are sensitive to the secondary structure and flexibility of the polypeptide. Structuring-induced chemical shift changes (observed shifts minus random coil reference values) were analyzed using the CSDb algorithm available at https://andersenlab.chem.washington.edu/CSDb/ (Eidenschink et al., 2009; Fesinmeyer, Hudson & Andersen, 2004). Figures 1A–1C show such plots for the three ligands. For Schgr-AKH-II both H^N^ and H_α_ are shifted up-field, while for Locmi-AKH-I and Aedae-AKH, only the H^N^ deviations are up-field. The H_α_ deviations are small and random. It has been shown that polypeptides with a helical structure have, H^N^ and H_α_ chemical shifts which are, on average, −0.30 ppm less than their random coil values, while β-sheet structures have shifts of ca. 0.6 ppm (Szilágyi, 1995).

**Table 2:** ^1^H chemical shifts of Schgr-AKH-II.

#	Res	HN	Hαα	Hβ (Hβ′)	Others
**A. DPC micelle solution, pH 4.5, 20 mM phosphate, Temp = 280 K**					
1	Glu	7.742	4.361	2.335	Hγ:2.483, 1.951
2	Leu	8.259	4.232	1.614, 1.318	Hδ:0.863
3	Asn	8.364	4.660	2.767, 2.647	Hδ:6.864, 7.565
4	Phe	7.978	4.561	3.071, 2.992	
5	Ser	8.096	4.289	3.782, 3.689	
6	Thr	7.923	4.179	3.908	Hγ:1.040
7	Gly	8.145	3.845, 3.905		
8	Trp	7.790	4.587	3.181, 3.183	Hδ:7.164, Hɛ:9.894, 7.513, Hζ:7.360, 6.959, Hη:7.005
**B. 30% DMSO solution, pH 4.5, 20 mM phosphate, Temp = 298 K**					
1	Glu	7.770	4.173	2.238	Hγ:2.361, 1.888
2	Leu	8.199	4.160	1.436, 1.304	Hδ:0.732, 0.780
3	Asn	8.208	4.501	2.519, 2.594	Hδ:6.777, 7.440
4	Phe	8.077	4.467	3.046, 2.849	
5	Ser	8.134	4.318	3.710, 3.667	
6	Thr	7.917	4.165	4.076	Hγ:na, 1.050
7	Gly	8.132	3.794, 3.691		
8	Trp	7.874	4.466	3.187, 3.018	Hδ:7.096, Hɛ:10.111, 7.539, Hζ:7.355, 7.021, Hη:7.103

**Table 3:** ^1^H and ^13^C chemical shifts of Locmi-AKH-I. ^1^H and ^13^C chemical shifts of Locmi-AKH-I in DPC micelle solution at 285 K, a pH of 5.0 and 20 mM phosphate buffer.

#	Res	HN	H_α_ (C_α_)	H_β_ (H_β′_) (C_β_)	Others
1	Glu		4.09	1.642	Hγ:1.541, 1.145
2	Leu	8.271	4.32 (54.7)	1.501, 1.715 (41.8)	Hγ:1.620, Hδ:0.978, 0.964 Cδ1:23.4, Cδ:2 25.0
3	Asn	8.491	4.71	2.715, 2.806 (38.6)	Hδ:6.871, 7.585
4	Phe	7.768	4.67 (52.3)	2.984, 3.072 (40.3)	Hδ:7.182, Hɛ:7.260, Hζ:7.289 Cδ:131.5, Cɛ:130.8, Cζ:130.6
5	Thr	7.987	4.49 (57.8)	4.015 (69.9)	Hγ:1.080 Cγ2:20.7
6	Pro		4.44 (59.0)	2.031, 2.586 (27.8)	Hγ:2.418 Cγ:31.9
7	Asn	8.336	4.68	2.672, 2.801 (35.6)	Hδ:6.851, 7.563
8	Trp	7.529	4.74	3.301 (29.7)	Hδ:7.247, 7.091, Hζ:7.448, 7.617, Hη:7.130 Cδ:126.4, Cɛ:121.1, Cη:123.6, Cζ:113.5, 120.2
9	Gly	8.316	3.94, 4.05 (45.3)		
10	Thr	7.988	4.288 (61.5)		Hγ:1.207, Cγ:21.3

**Table 4:** ^1^H chemical shifts of Aedae-AKH. ^1^H chemical shifts of Aedae-AKH in DPC micelle solution at 285 K, a pH of 5.0 and 20 mM phosphate buffer.

#	Res	HN	HA	HB (HB′)	Others
1	Glu		4.41	2.36	Hγ:2.53, 1.962
2	Leu	8.26	4.38	1.47, 1.66	Hγ:1.68, Hδ:0.91, 0.932
3	Thr	8.20	4.33	4.18	Hγ:1.10
4	Phe	7.82	4.66	3.28	Hδ:7.15, Hɛ:7.22, HZ:7.25
5	Thr	7.96	4.42	3.93	Hγ:1.02
6	Pro		4.42	1.44, 1.56	Hγ:1.97, 2.53, Hδ:3.08, 3.38
7	Ser	8.28	4.25	3.81	
8	Trp	7.82	4.63	2.93, 3.04	Hδ:7.21, Hɛ:9.99, 7.06, Hζ:7.40, 7.58, Hη:7.09

Thus, each of these peptides has a β-turn structure. Similar results were found for a number of other deca- and octapeptidic members of the AKH family, i.e., for Declu-CC, Melme-CC, and Dappu-RPCH (Jackson et al., 2018). However, downfield shifts were previously found for Anoga-HrTH from the *Anopheles* mosquito (Mugumbate, Jackson & Van Der Spoel, 2011; Mugumbate et al., 2013).

Using the Random Coil Index tool (Tremblay, Banks & Rainey, 2010), the chemical shifts were also used to estimate the model-free order parameter, *S*^2^, of the peptides (see Fig. 1D). An order parameter of 1 means the peptide is rigid, while an order parameter of 0 means the peptide has no structure. Figure 1D shows that Locmi-AKH-I is very ordered, with a maximum order parameter of 0.9 around proline, whereas the C-terminal has less ordering (*S*^2^ = 0.30). On the other hand, Schgr-AKH-II and Aedae-AKH are much more flexible. The order parameter for these two peptides range from 0.1 to 0.4, which is similar to that of Dappu-RPCH, an AKH peptide member from the crustacean water flea (Jackson et al., 2018).

### MD simulation with DPC micelle

**Figure 2:**
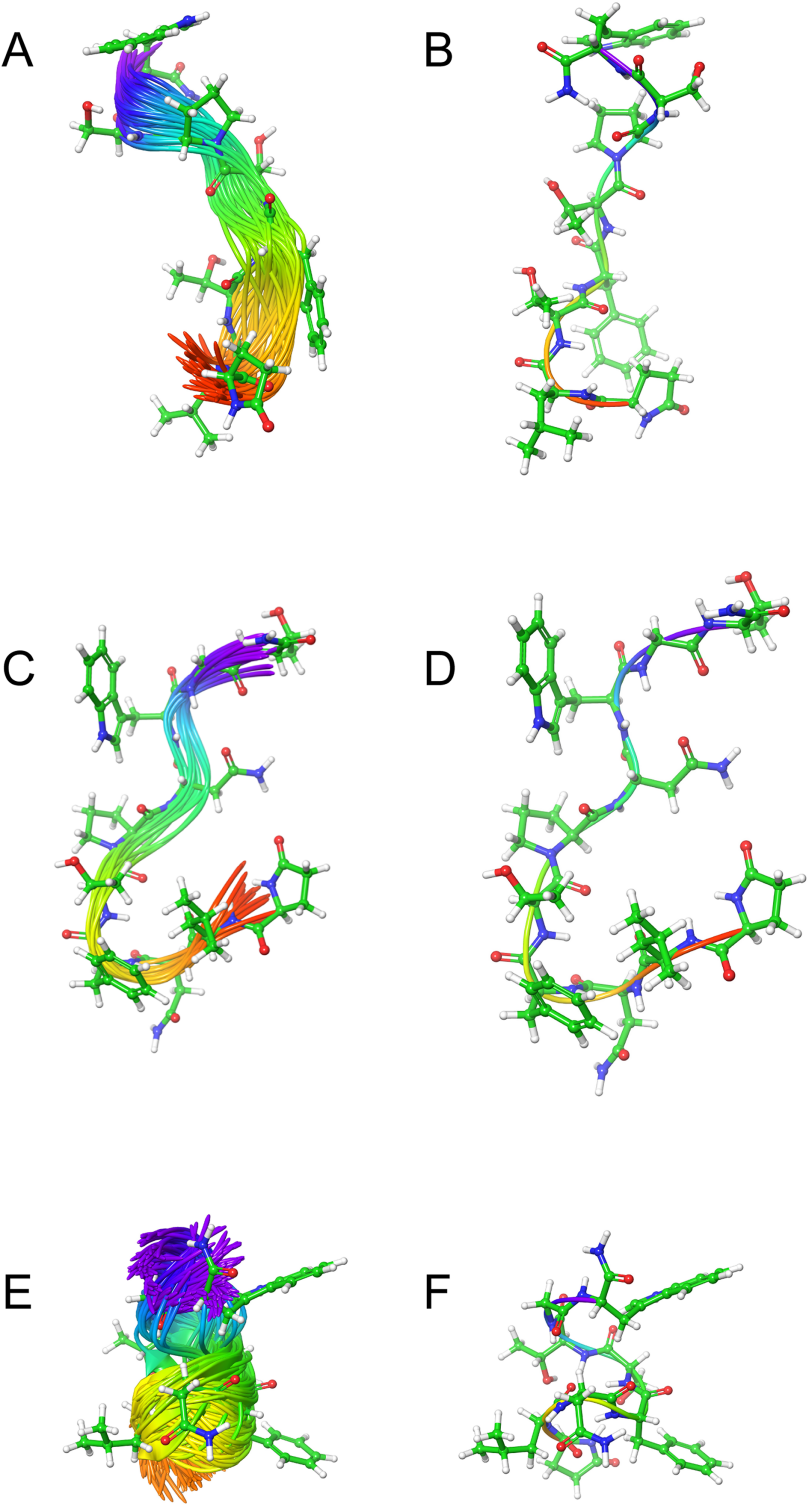
Solution cluster overlay and the root conformation of Schgr-AKH-II, Locmi-AKH-I, and Aedae-AKH in DPC micelle solution. (A) Cluster overlay of Locmi-AKH-I, (B) root conformer of Locmi-AKH-I, (C) cluster overlay of Aedae-AKH-I, (D) root conformer of Aedae-AKH-I, (E) cluster overlay of Schgr-AKH-II, and (F) root conformer of Schgr-AKH-II.

**Figure 3:**
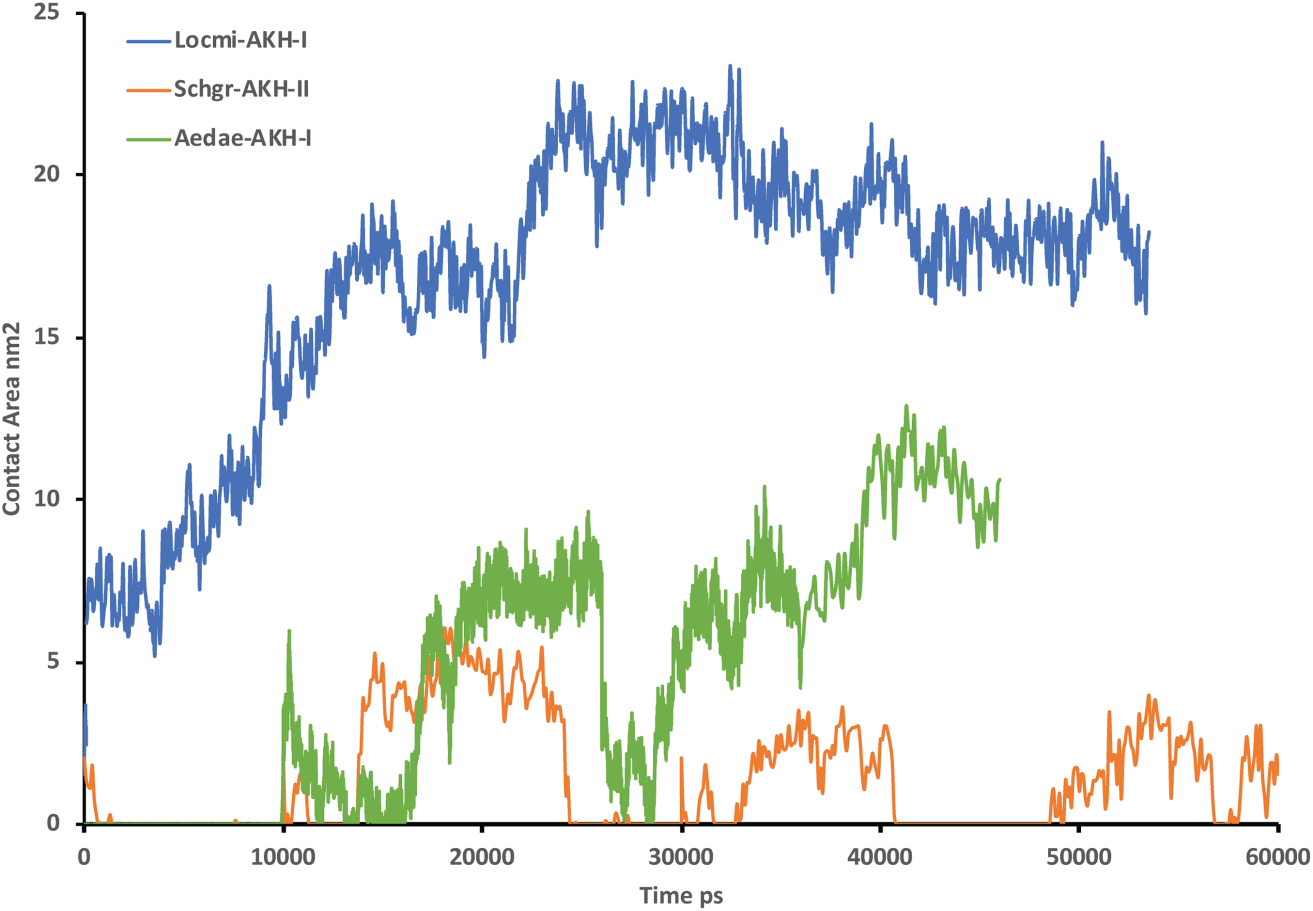
Contact surface area between the peptides and DPC micelle. Plot of contact area between peptide and a DPC micelle during a 60 ns simulation of Locmi-AKH-I, Aedae-AKH-I, and Schgr-AKH-II, as a function of time.

Figure 2 shows the solution structures of the three AKH ligands in DPC micelle solution. In each case, the MD was started with the peptide in water, but they rapidly diffused to interact with the DPC micelle. Depending on the starting orientation of the peptide relative to the micelle, the peptide would make contact with the phospholipid and move away until a stable orientation was established. This is shown in Fig. 3 where the peptide/DPC contact area is plotted as a function of time. For Locmi-AKH-I, contact between the DPC and micelle is established during the equilibration period, for Aedae-AKH it is established after 30 ns, while for Schgr-AKH-II, even after 60 ns the peptide is still not permanently attached to the micelle. It is interesting to note that the contact area between Locmi-AKH-I is much higher than that for the other two peptides and Locmi-AKH-I is much more rigid even though it is longer, a decapeptide versus an octapeptide. The interaction between the peptides and the lipid surface, as shown by the contact area, is important as it has been postulated that, before the ligand binds to its receptor, it first binds to the cell membrane surface. Thus, surface binding is an important step in receptor activation.

In order to show how the structure of the peptides changed during the MD, an overlay of each peptide is shown in Figs. 2A–2C. Cluster analysis of the trajectory gave a single large cluster for each peptide with a number of smaller, high energy, clusters. The root conformer and an overlay of each cluster is shown in Fig. 2. The predominant conformation of each peptide does have a turn feature but the details differ for each AKH. Locmi-AKH-I (Fig. 2A) has a clear β-turn around its proline residue; Aedae-AKH (Fig. 2B) has a more open structure compared to the other peptides and no marked turn around proline; Schgr-AKH-II (Fig. 2C) is tightly coiled in DPC solution.

### Receptor construct

Use of the GPCR-ModSim server gave the crystal structure of the hĸ-OR (PDB ID: 4DJH), with 2.9 Å resolution, as a top template for Schgr-AKHR. The crystal structure has the selective antagonist JDTic complexed to the receptor (Wu et al., 2012). This template has the highest sequence identity (26.33%) compared to other templates as shown in Fig. S2. hĸ-OR belongs to the class A (rhodopsin-like), γ-subfamily of GPCRs and was selected to build models for Schgr-AKHR. The initial sequence alignment from the GPCR-ModSim server was aligned manually with the use of Chimera (Pettersen et al., 2004). The predicted TM helices of Schgr-AKHR and the PDB structural assignments of hĸ-OR were also used to confirm the alignment as shown in Fig. S1. The sequence analysis shows that the conserved residues in the seven TM helices (TM1–TM7) of hĸ-OR are also highly conserved within Schgr-AKHR (conserved residues highlighted by purple colored boxes in Fig. S1), indicating that they may be involved in functions, such as signaling and ligand binding, of Schgr-AKHR. Additionally, as in hĸ-OR, disulfide forming cysteine residues (Cys-131 and Cys-210) are conserved in Schgr-AKHR. The predicted TM helices of Schgr-AKHR (Fig. S3) are consistent with the TM helices of hĸ-OR: there are no gaps or insertions in these regions, signifying that the target sequence is correctly aligned with the template sequence and, hence, can be used for the modeling process.

The homology models of Schgr-AKHR were built using the *Modeler* 9v7 program. The input parameters of *Modeler* were set to generate 100 models with high structural optimization. A disulfide bridge, Cys-131–Cys-210, was defined as in the template structure. The best model was selected based on the lowest PDF (molecular probability density function) energies and DOPE score (discrete optimized protein energy) for the docking simulations. The selected models' qualities were subsequently assessed with structural evaluation programs such as PROCHECK and ERRAT. Ramachandran plot analyses of the Schgr-AKHR model from PROCHECK are shown in Fig. S4. All the residues were either in favored or in allowed regions, indicating that the backbone torsion angles (phi and psi) of this model are reliable. In addition, the ERRAT score, so-called overall quality factor, was computed on the Schgr-AKHR model to check the quality of its non-bonded atomic interactions. The normally accepted score range for a high-quality model is >50 (Colovos & Yeates, 1993). The ERRAT score for the Schgr-AKHR model was 78, showing that the model is within the high-quality range. All these validation methods demonstrated that the model is reliable and can be used for further studies. The final 3D structure of the Schgr-AKHR model, as shown in Fig. S5A, is similar in overall fold with the template protein hĸ-OR (Fig. S5B). The superposition of the Schgr-AKHR model with that of hĸ-OR displayed a 2.804 Å root mean squared deviation (RMSD) for 1,332 atoms pairs. This low RMSD demonstrates that the overall tertiary structure of the model is similar to the template structure. In addition, the superimpositions indicate that the seven TM helices are highly conserved with hĸ-OR. However, there are slight structural variations in the loop regions of the model.

### Ligand docking

**Figure 4:**
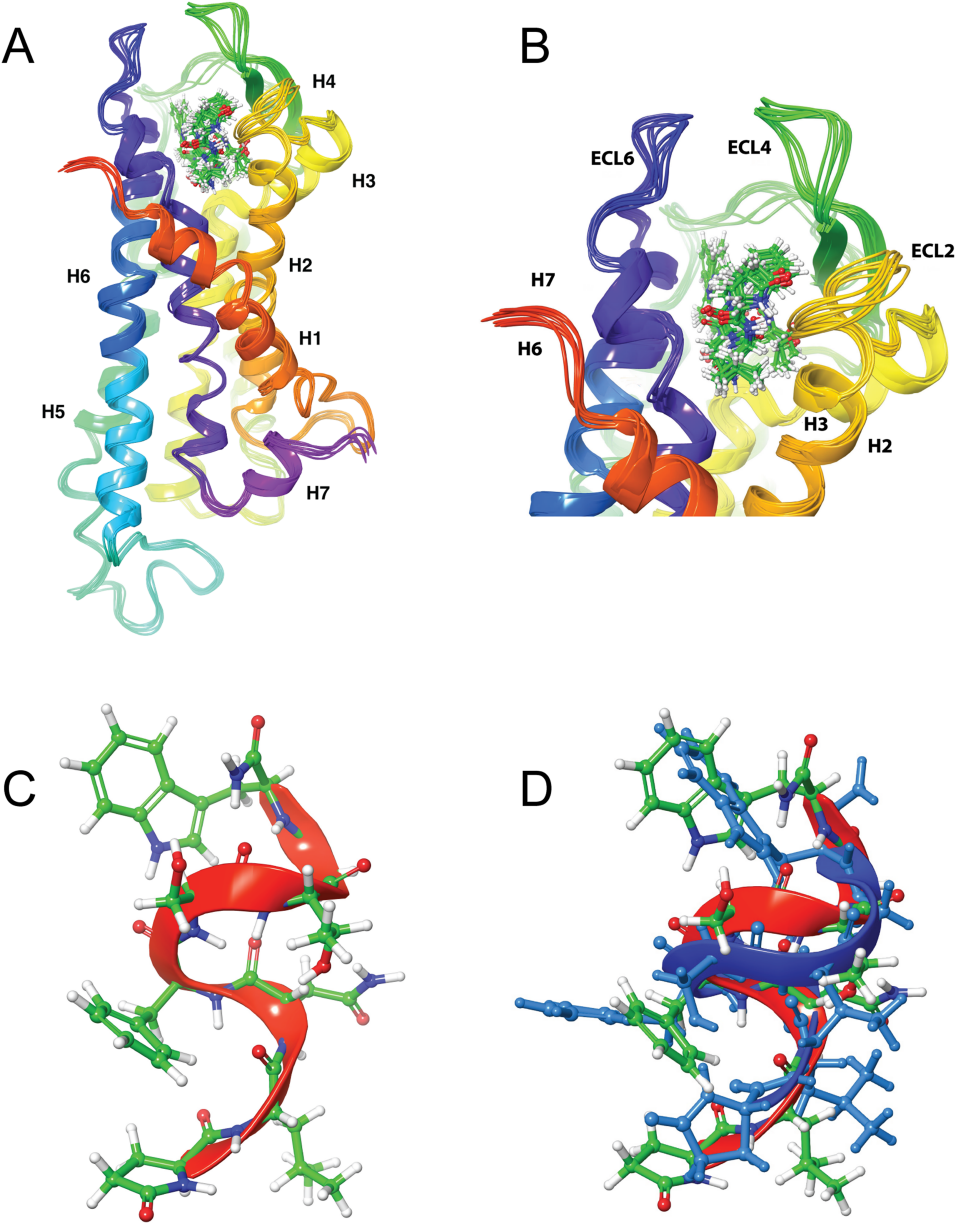
Structure of Schgr-AKH-II in solution and bound to Schgr-AKHR. (A) Overlay of several snapshots of the dynamic simulation of Schgr-AKH-II in the Schgr-AKH receptor binding pocket. Helices are labeled H1–7. (B) Enlargement of binding pocket showing orientation of peptide. Important extracellular loops are labeled. (C) Conformation of bound Schgr-AKH-II. (D) Overlay of best binding pose and root conformer from simulation in DPC micelle solution.

**Figure 5:**
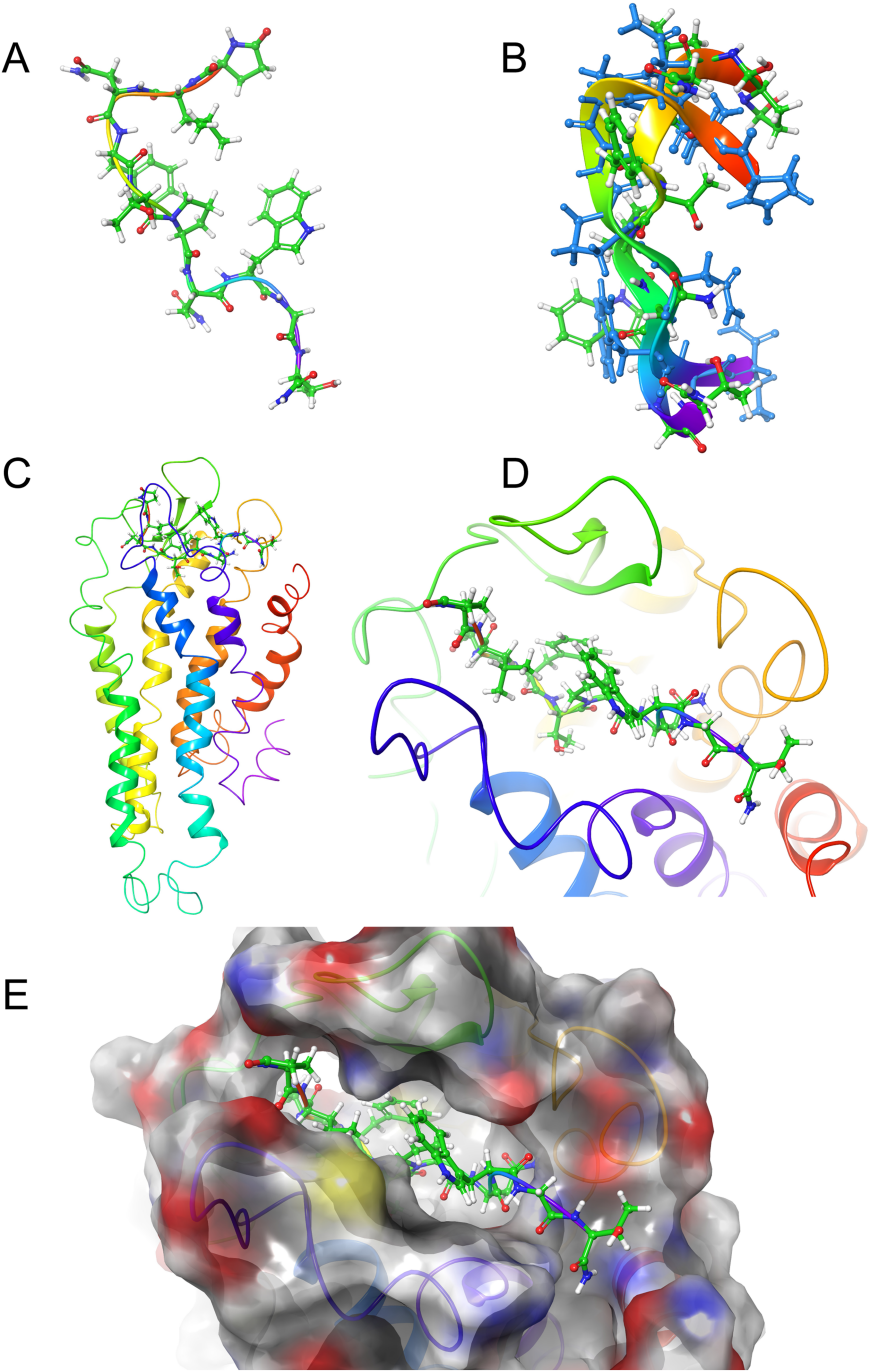
Structure of Locmi-AKH-I in solution and bound to Schgr-AKHR. (A) Conformer of bound Locmi-AKH-I, (B) overlay of bound Locmi-AKH-I (blue) and in DPC micelle solution, (C) pictorial representation of Schgr-AKHR with Locmi-AKH-I in the binding pocket, (D) enlargement of binding pocket showing the orientation of the peptide, and (E) binding pocket surface with Locmi-AKH-I inserted. Surface is colored according to electrostatic potential, red for negative potential, blue for positive potential, and gray for neutral potential.

**Figure 6:**
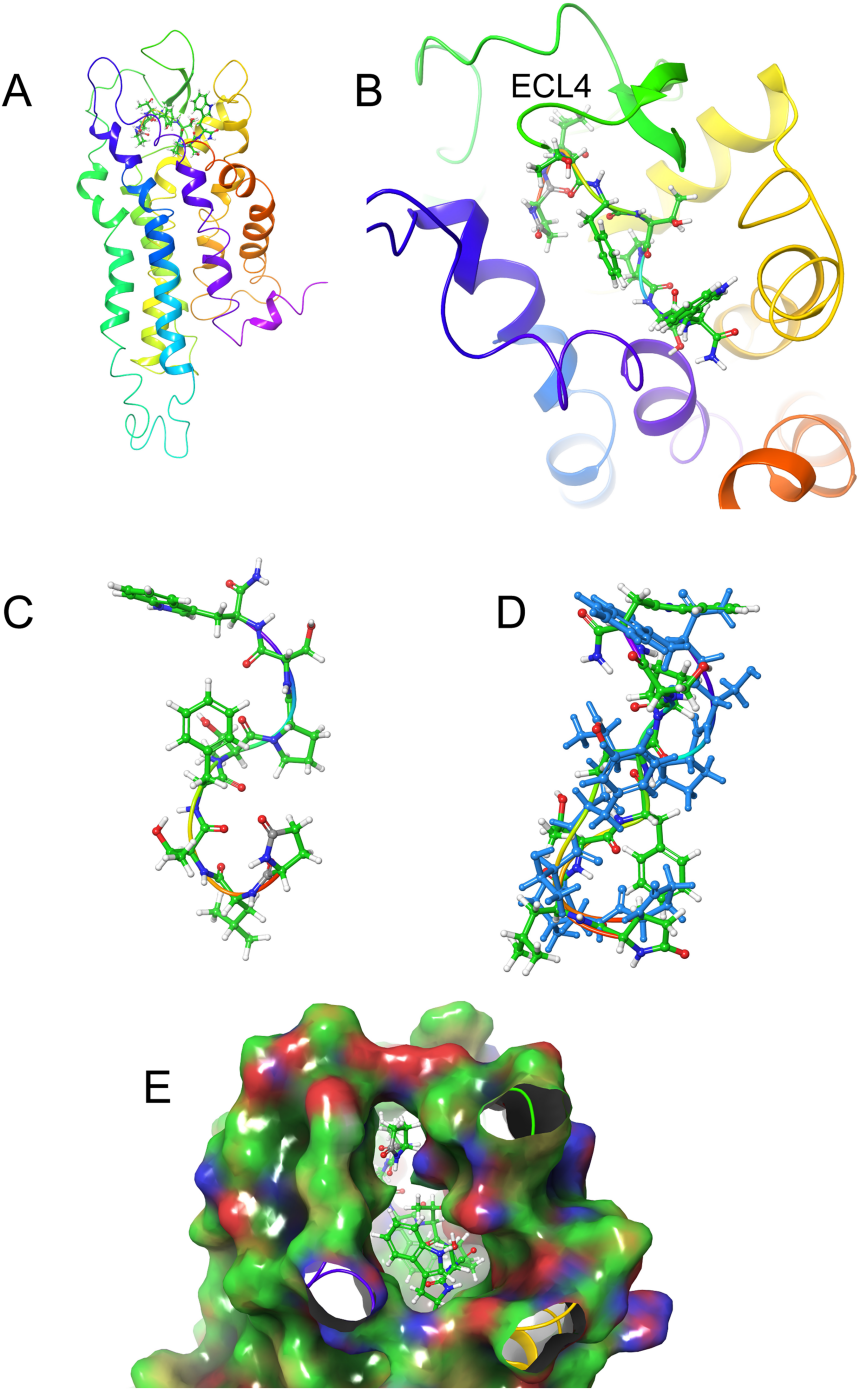
Structure of Aedae-AKH in solution and bound to Schgr-AKHR. (A) Pictorial representation of Schgr-AKHR with Aedae-AKH in the binding pocket, (B) enlargement of binding pocket showing the orientation of peptide, (C) conformer of bound Aedae-AKH, (D) overlay showing the different conformers of bound Aedae-AKH (blue) and in DPC solution, and (E) binding pocket surface with Aedae-AKH inserted. Surface colored according to electrostatic potential, red for negative potential, blue for positive potential, and green for neutral potential.

LigPrep was used to generate multiple conformers of the three AKH peptides, which were then docked to the Schgr-AKHR model. A receptor grid was generated for the extra-cellular half of the GPCR and the peptide docked using SP-Peptide precision. A total of 100 different poses were collected and scored for each peptide. An overlay of the highest scoring poses gave the same receptor binding site for all three peptides. This binding pocket consisted of a cleft running across the top of Schgr-AKHR, between helices 2, 6, and 7 and extra-cellular loops 2 and 4 (details shown in Figs. 4–6). The peptides lay along this cleft. It was found that, while the GLIDE protocol tried to dock many different conformations of the peptide, only those with some turn structure were successful. Many of the docked poses had similar GLIDE scores but different sets of peptide/receptor interactions. During the docking, it was found that the orientation of the peptide within the binding pocket did not change. For this reason, the docking was repeated with the peptide rotated through 180° around the axis of the TM helices, i.e., the direction of N-terminus to the C-terminus was reversed. Again, the peptide could be docked successfully, indicating that the binding pocket was quite promiscuous. Although either orientation of the peptide would bind to the receptor, the binding energies of the two orientations differed by some 50 kcal/mol and hence the original orientation was chosen for further study.

The docked structure of each AKH peptide with the highest binding energy, was used as the starting structure for MD of the complex in a POPC membrane. During the dynamics, the ligands were found to move, and individual side-chains rotate within the binding site, making and breaking H-bonds to various residues of the receptor. This was essentially the same as what was found during the GLIDE docking. While the POPC membrane added to the computational cost of the simulation, it was necessary to prevent the transmembrane helices of the receptor from moving apart. Snapshots of the simulation were transferred to Maestro, where Prime-MM-GBSA was used to calculate the binding energy.

Figures 4A and 4B shows an overlay of several snapshots of the dynamic simulation of the octapeptide, Schgr-AKH-II, in its receptor binding pocket. As can be seen, the overall conformation of Schgr-AKH-II remains the same (Fig. 4C) but the side-chains move within the binding site, forming and breaking intra- and inter-molecular H-bonds. There is also some movement of the receptor during the dynamics. The free energy of binding of the different snapshots were not significantly different and ranged from −94 to −116 kcal/mol over the 1 μs simulation.

The bound conformation of Schgr-AKH-II is shown in Fig. 4C, while Fig. 4D is an overlay of bound Schgr-AKH-II and its lowest energy conformation in DPC micelle solution. The agreement of these two conformers is remarkable, especially considering that the GLIDE—SP protocol generates some 100 different starting conformations for the peptide docking.

Figure 5A shows the bound conformation of Locmi-AKH-I, while Fig. 5B is an overlay of this bound conformer and the lowest energy conformer found in DPC micelle solution. The agreement here is not as close as that for Schgr-AKH-II, but the same turn structure is seen. Figure 5C shows Locmi-AKH-I in the receptor binding pocket, while Fig. 5D and 5E show the details of how Locmi-AKH-I fits into the receptor: the decapeptide stretches across the cleft in the receptor with the central portion of the peptide fitting into the binding pocket, but the two termini pointing outside the binding pocket. Locmi-AKH-I gave the most trouble during the docking stage as poses were frequently rejected. During the MD, the terminal amide of this decapeptide was sometimes found to H-bond to a POPC molecule, which is of course not present during the GLIDE docking. The final binding energy for Locmi-AKH-I was −98 kcal/mol.

Figures 6A and 6B show how Aedae-AKH fits into the binding pocket of the Schgr-AKHR model. The arrangement is similar to that of Locmi-AKH-I, except, in this case, the termini do not extend outside the receptor. In the case of Aedae-AKH, ECL4 folds over the top of the binding site, trapping the peptide inside. The conformation of bound Aedae-AKH is shown in Fig. 6C, and an overlay with the DPC micelle solution conformation is shown in Fig. 6D. Again, these two conformations are very similar, supporting the idea that the peptides are pre-arranged on the cell surface. The binding energy of Aedae-AKH was −88 kcal/mol.

**Figure 7:**
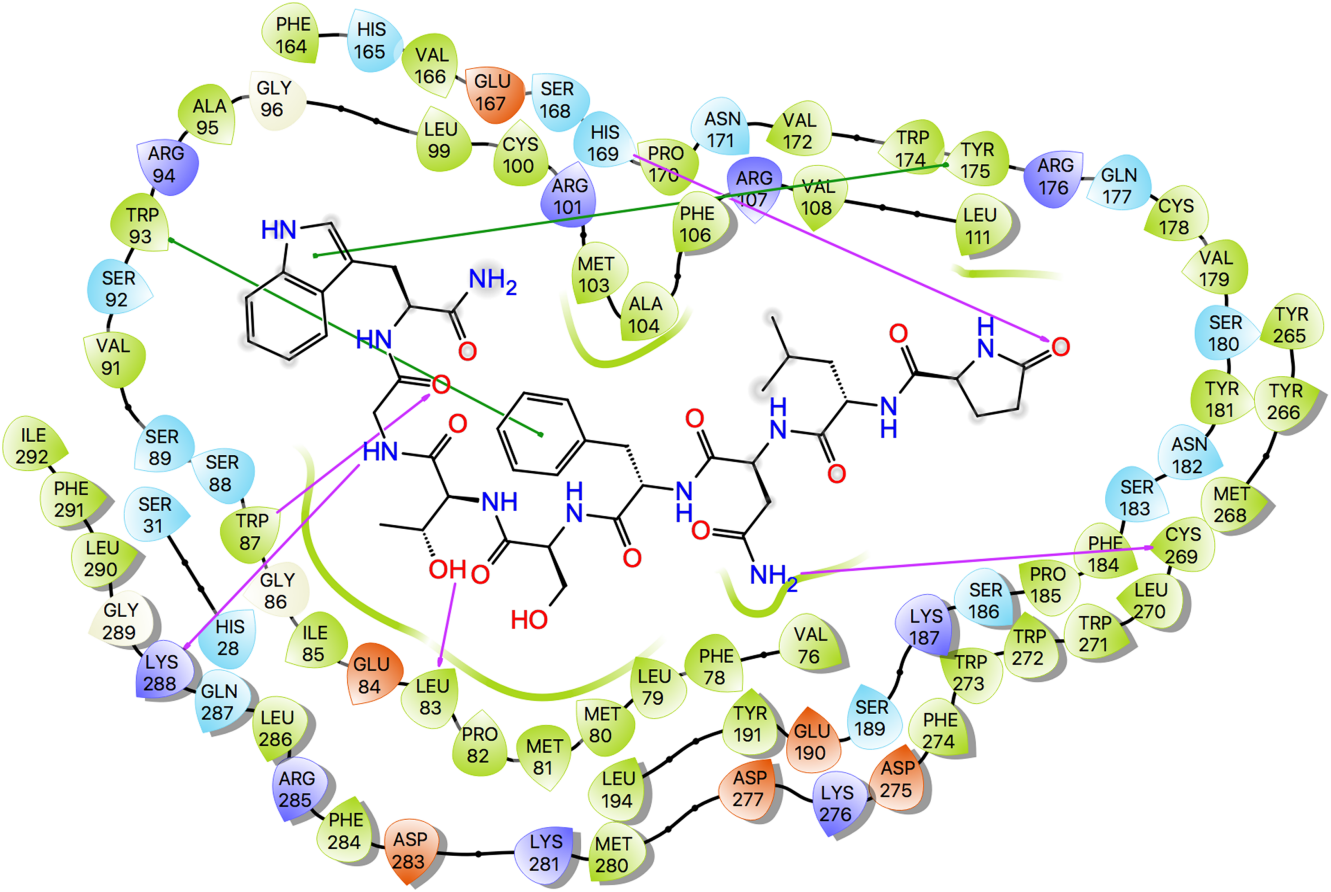
Protein-ligand interaction diagram, Schgr-AKHR - Schgr-AKH-II. Residues are represented as colored spheres, labeled with the residue name and residue number, and colored according to their properties, green for hydrophobic, blue for positive charge, and red for negative charge. The ligand is displayed as a 2D structure. Interactions between the residues and the ligand are drawn as lines, colored by interaction type, purple for H-bonding and green for pi–pi stacking. The binding pocket is indicated by a line drawn around the ligand, colored by the color of the nearest residue. Solvent exposure is indicated on the ligand atoms, and by the break in the line drawn around the pocket.

Ligand interaction diagrams for the three ligands are shown in Fig. 7, while Table 5 lists the interactions between the ligands and Schgr-AKHR. From these data, it is clear that all three AKH ligands have very similar interactions with Schgr-AKHR. Both Schgr-AKH-II and Aedae-AKH, H-bond to His169 of the receptor, while W^8^ of Locmi-AKH-I π-stacks with this residue. In Locmi-AKH-I, the amide carbonyl, pE^1^CO, H-bonds to Lys281, while in Aedae-AKH it is pE^1^O_ɛ1_ which H-bonds to Lys281. The terminal residue of both Schgr-AKH-II and Locmi-AKH-I H-bond to Lys288.

**Table 5:** List of interactions between peptide and receptor in the binding pocket of Schgr-AKHR.

Schgr-AKH-II		Locmi-AKH-I		Aedae-AKH	
Ligand	Receptor	Ligand	Receptor	Ligand	Receptor
pE^1^O_ɛ1_	His169	pE^1^CO	Lys281	pE^1^CO	His169
N^3^NH_2_	Cys269	N^3^CO	Trp273	pE^1^O_ɛ1_	Lys281
F^4^π–π stack	Trp93	T^5^OH	Gln287	S^7^OH	Cys178
S^5^OH	Ser92	N^7^CO	Trp87	S^7^NH	Cys178
G^7^NH	Lys288	N^7^NH_2_	Ser92	W^8^H_ɛ1_	Ser180
G^7^CO	Trp87	T^10^CO	Lys288	W^8^NH_2_	Ser183
W^8^CO	Lys288	W^8^π–π stack	His169		
W^8^π–π stack	Tyr175	T^10^NH_2_	His28		

### Analysis of molecular switches

A feature of class A GPCRs is the presence of highly conserved molecular switch motifs. These switches, which play key roles in the stabilization of the receptor in an inactive and active state, include a TM3–6 lock, a SPLF switch, a tyrosine toggle, and a DRY ionic lock. The breaking of these switches results in movement of the TM helices, which can activate the receptor (Trzaskowski et al., 2012). These switches are the same as those reported for the AKHR of *Drosophila melanogaster*, *Tribolium castaneum, Anopheles gambiae*, and *Rhodnius prolixus* (Rasmussen et al., 2015), suggesting that the activation mechanism of Schgr-AKHR may be the same.

**Figure 8:**
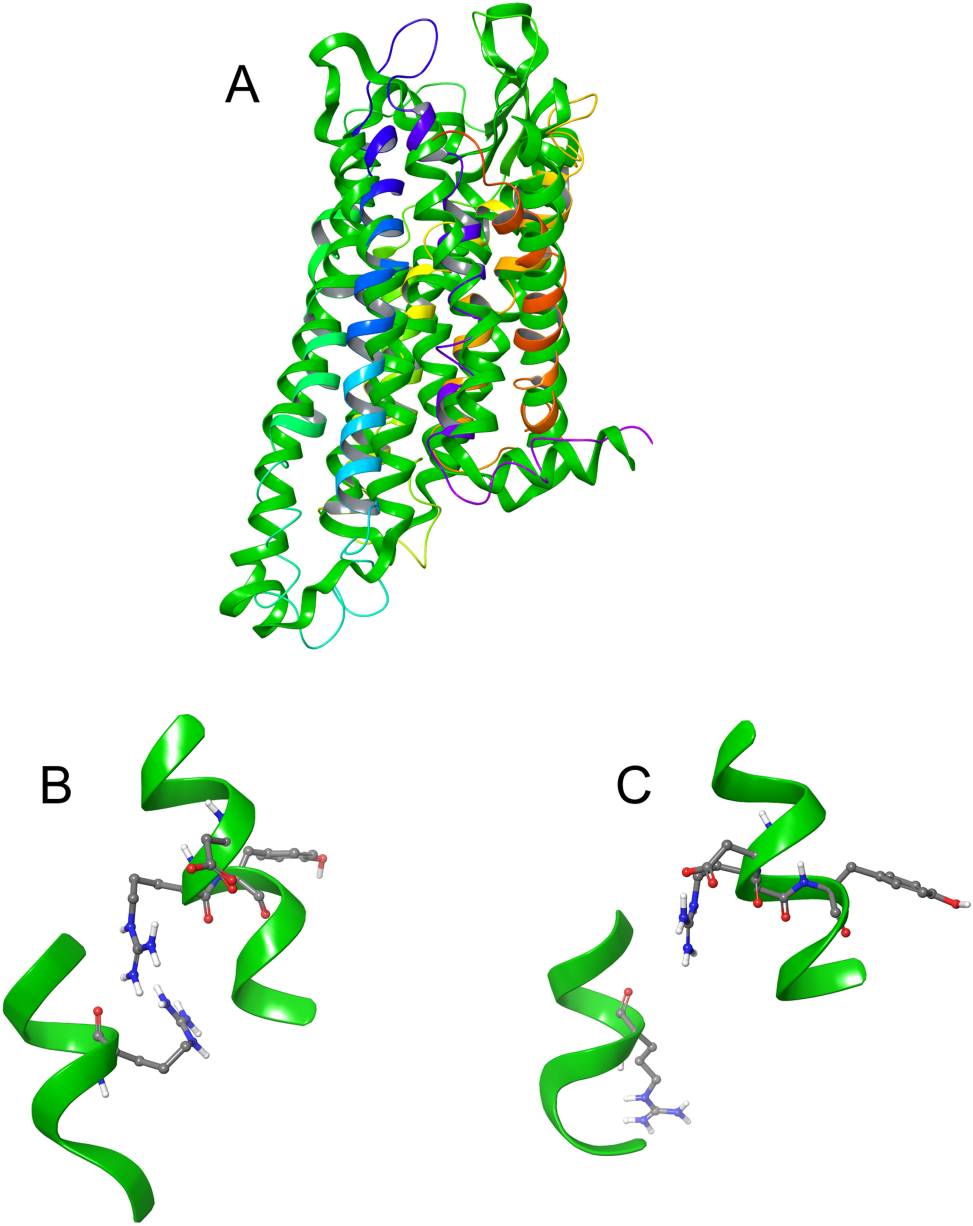
Comparison of active and inactive Schgr-AKH-II receptor. (A) Overlay of active (colored) and inactive (green) *S. gregaria* receptor, (B) closed DRY switch, and (C) open DRY switch. Only polar hydrogens are shown.

The DRY ionic lock between arginine and tyrosine is postulated to open and close during receptor activation. This is shown in Fig. 8. In the inactive state, the DRY switch is closed (Fig. 8B) but upon ligand binding, TM6 and TM3 twist, opening this switch (Fig. 8C).

**Figure 9:**
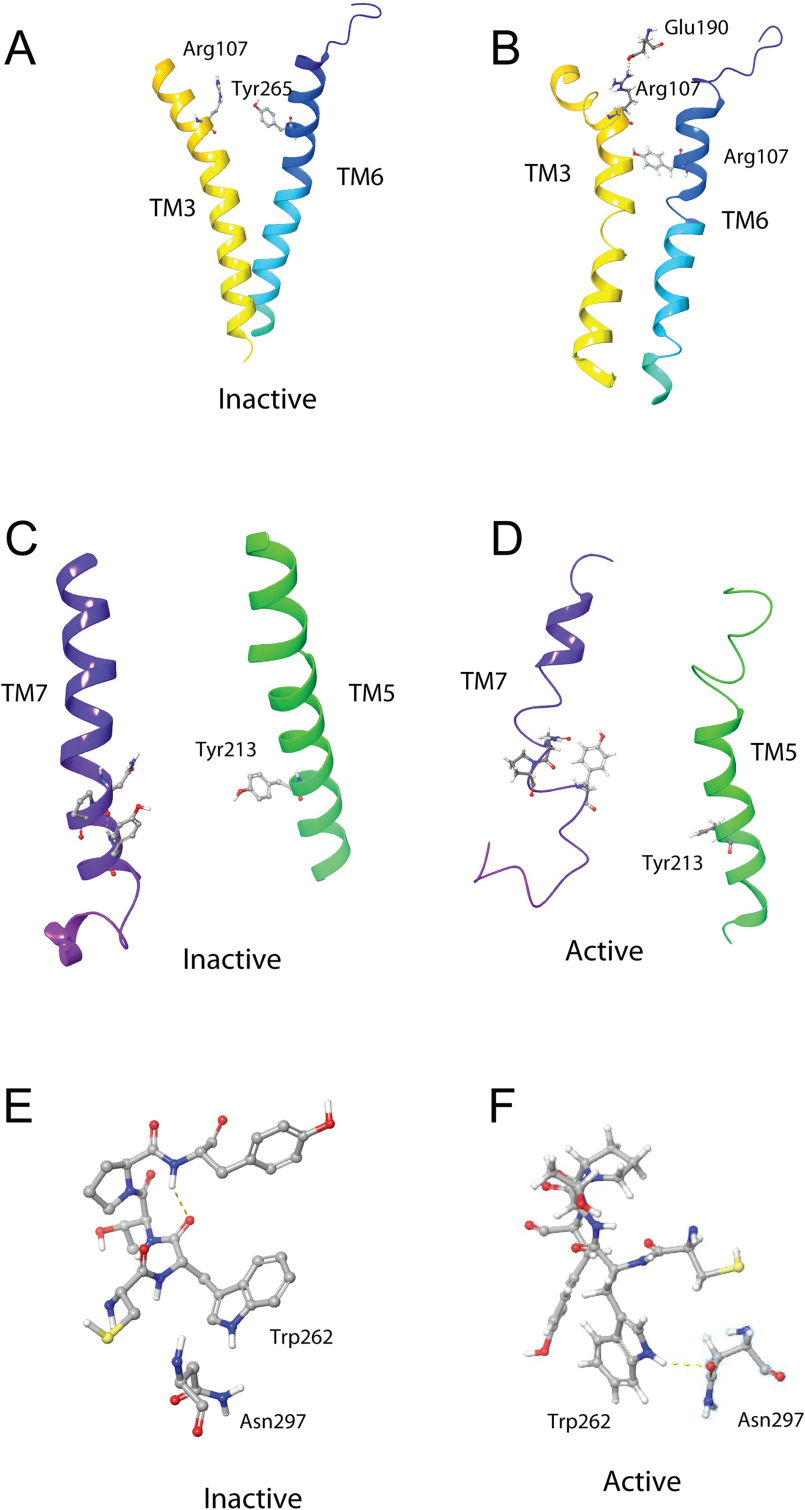
Schgr-AKHR molecular switches of the active and inactive receptor. (A) TM3–6 lock of inactive receptor, (B) TM3–6 lock of active receptor, (C) tyrosine toggle switch of inactive receptor, (D) tyrosine toggle switch of active receptor, (E) CWxPY motif on TM6 of inactive receptor, and (F) CWxPY motif on TM6 of active receptor.

The TM3–6 lock involves two residues in the binding pocket, Arg^107^ on TM3 and Tyr^265^ on TM6 (Fig. 9A). In the inactive state these two residues are far apart, but on ligand activation, they move closer together. Figure 9A also shows, in the active state, Arg^107^ H-bonding with Glu^190^ of ECL4. It was noted before that this loop closes over the binding pocket after ligand binding. In Fig. 9A one can also clearly see how TM6 and TM3 move together on the extra-cellular side but move away from each other on the intracellular side, upon activation.

The tyrosine toggle switch involves the NPxxY motif on TM7 and Tyr^213^ on TM5 (Fig. 9B).

## Discussion

As has been found before, insect neuropeptides are flexible in solution but generally have a preferred β-turn conformation (Mercurio et al., 2018; Shen et al., 2018; Zubrzycki, 2000). Using CD spectroscopy, Cusinato et al. (1998) proposed a P II extended conformation for the AKH/RPCH peptides, at low temperatures, in aqueous solution. However, they found that the majority of AKH/RPCH peptides adopted a β-turn conformation in the presence of 0.6% SDS. This is what was found in DPC micelle solution by both NMR chemical shift and molecular modeling results for the three peptides Schgr-AKH-II, Aedae-AKH, and Locmi-AKH-I in the current study. These peptides are not very soluble in water but are readily soluble in DPC micelle solution, which is a clear indication that they interact with the micelle. Previously, using DOSY NMR spectroscopy, we showed that the crustacean AKH member, Dappu-RPCH, binds to the micelle in DPC solution, and that the micelle consists of ~50 DPC molecules (Jackson et al., 2018). This was also shown by the molecular modeling for the three locust peptides in the present study. Locmi-AKH-I interacted strongly with the micelle and perhaps this is the reason why this peptide had a much higher order parameter than the other two peptides.

Despite chain length and sequence differences, all three peptides were found to have the same binding site on the receptor. They all had similar binding constants and interacted with the same receptor residues. This is in agreement with previous results of Marchal et al. (2018) where many members of the AKH family activated Schgr-AKHR in vitro, to the same extent and it was concluded that this receptor was quite promiscuous. In the present study, multiple binding poses were found for the peptides in the binding site and this was confirmed by MD in a POPC membrane. Here each peptide was found to move within the binding site, interacting with different residues. This may account for the same receptor being activated naturally by all three peptides. Interestingly, during the MD, the receptor itself moved, closing over the binding site and opening up on the intra-cellular side. This motion has been postulated to result in receptor activation, with a G-protein able to bind to the more open receptor. Figure 8A, which is an overlay of the active and inactive receptor, shows this movement of the helices. Measurements show that Ala-243, on TM6, moves some 6.4 Å on receptor activation. One can also see that ECL6 and ECL4 (Fig. 4B) close over the binding site.

It is interesting to compare and contrast the binding of Schgr-AKH-II, Aedae-AKH, and Locmi-AKH-I with the binding of a crustacean RPCH from *Daphnia pulex*, Dappu-RPCH, to its cognate receptor, Dappu-RPCHR (Jackson et al., 2018). Both receptors have similar binding sites involving TM2, 6, and 7 but Dappu-RPCHR also uses extracellular loops 1, 2, and 3, while Schgr-AKHR involves loops 2 and 4 (and loop 6 in the active receptor). Dappu-RPCH undergoes significant conformational changes upon receptor binding, having a more extended structure in solution but a more pronounced β-turn when bound. On the other hand, Schgr-AKH-II, Aedae-AKH, and Locmi-AKH-I, undergo very little conformational change upon receptor binding. This might account for the higher binding constant of these three peptides relative to Dappu-RPCH. The similarity between the AKH/RPCH systems is understandable given the evolution of the AKH/corazonin/ACP/GnRH receptor superfamily and their ligands (Hauser & Grimmelikhuijzen, 2014).

The presented model of Schgr-AKHR can be compared to another class A GPCR, the human gonadotropin releasing hormone receptor, GnRHR (Flanagan & Manilall, 2017). This receptor has an exaggerated bend around Pro, in a CWxPY motif found on TM6. In GnRHR, this bend is stabilized by a water mediated H-bond between Cys^47^ and Tyr^51^ on TM6 of the CWxPY motif and by H-bonding to a residue in TM7. The presented model of Schgr-AKHR also has this CWxPY motif in TM6, which results in a proline kink. In the active receptor, Cys and Tyr residues are correctly oriented for water mediated H-bonding, with a distance of 3.18 Å between them. Also, Trp^262^ of the CWxPY motif was found to H-bond to Asn^297^ of TM7. The functional importance of this motif was demonstrated by mutations associated with congenital hypogonadotropic hypogonadism. This rare disorder results from decreased production or secretion of gonadotropin-releasing hormone (GnRH) and/or lack of action of GnRH upon GnRHR. There are some 25 genes identified in this condition, but if the Pro residue is substituted by Arg, there is complete disruption of the receptor function. This shows the importance of this proline kink. On the other hand, when the Cys of this receptor motif is mutated to Tyr or Ala, the GnRH ligand does not bind to the receptor (Flanagan & Manilall, 2017). Interestingly, in the inactive Schgr-AKHR, there was a H-bond between Trp-CO and Tyr-NH but there was no H-bond between Trp^262^ and Asn^297^. Upon ligand binding, however, the helices moved in such a way that the one H-bond broke and the other formed. This is similar to the DRY switch described above. Binding similarities between GnRHR and Schgr-AKHR are not unexpected. It has long been accepted that GnRH and AKH are peptides belonging to the same superfamily because not only are the ligands structurally closely related but also the cognate receptors (Gäde, Šimek & Marco, 2011; Hauser & Grimmelikhuijzen, 2014; Roch, Busby & Sherwood, 2011, 2014).

## Conclusions

In this paper, we have shown that the putative receptor, Schgr-AKHR, is a member of the Class A superfamily of GPCRs. It has seven TM helices and the same conserved residues as other AKHRs. Schgr-AKHR also has a number of molecular switch motifs, which are a feature of class A GPCRs. Our results show that the three endogenous peptides, Schgr-AKH-II, Aedae-AKH, and Locmi-AKH-I, all bind to the same receptor binding site and with very similar binding constants. This may be surprising, as the three ligands are very different; their sequence is different and one is a decapeptide, while the other two are octapeptides. However, *L. migratoria* and *S. gregaria*, have only one AKHR so it could be expected that the three endogenous peptides would have to bind to this single receptor. These results also fit previous findings that, *in vitro*, the AKHR of *S. gregaria* is equally well activated by a number of AKH members.

The similarities in the ligand binding of Schgr-AKHR and Dappu-RPCH/Dappu-RPCHR supports the evolutionary development of the AKH/corazonin/ACP/GnRH receptor superfamily. The next step in this study would be to use *in silico* screening to identify suitable agonists or antagonists, which could act as next generation insecticides.

## Supplemental Information

10.7717/peerj.7514/supp-1 Supplemental Information 1 Supplemental figures. Details of homology modeling of Schgr-AKHR showing sequence alignment (Fig. S1), GPCR-ModSim server sequence identity (Fig. S2), Ramachandran analysis from PROCHECK (Fig. S3), and final 3D structure compared to the template structure hk-OR.

10.7717/peerj.7514/supp-2 Supplemental Information 2 Raw data for Schgr-AKH-II bound to Schgr-AKHR in PDB format.

## Additional Information and Declarations

### Data Availability

The following information was supplied regarding data availability: The coordinates of the *Schistocerca gregaria* receptor are available in Data S1.
