# Supplementary material for: The adipokinetic hormones and their cognate receptor from the desert locust, Schistocerca gregaria: solution structure of endogenous peptides and models of their binding to the receptor
Source: PeerJ. 2019 Aug 30;7:e7514. doi: 10.7717/peerj.7514 (PMC6718158; doi:10.7717/peerj.7514)
Supplement: Supplemental Information 1 — Details of homology modeling of Schgr-AKHR showing sequence alignment (Fig. S1), GPCR-ModSim server sequence identity (Fig. S2), Ramachandran analysis from PROCHECK (Fig. S3), and final 3D structure compared to the template structure hk-OR. [file peerj-07-7514-s001.pdf]

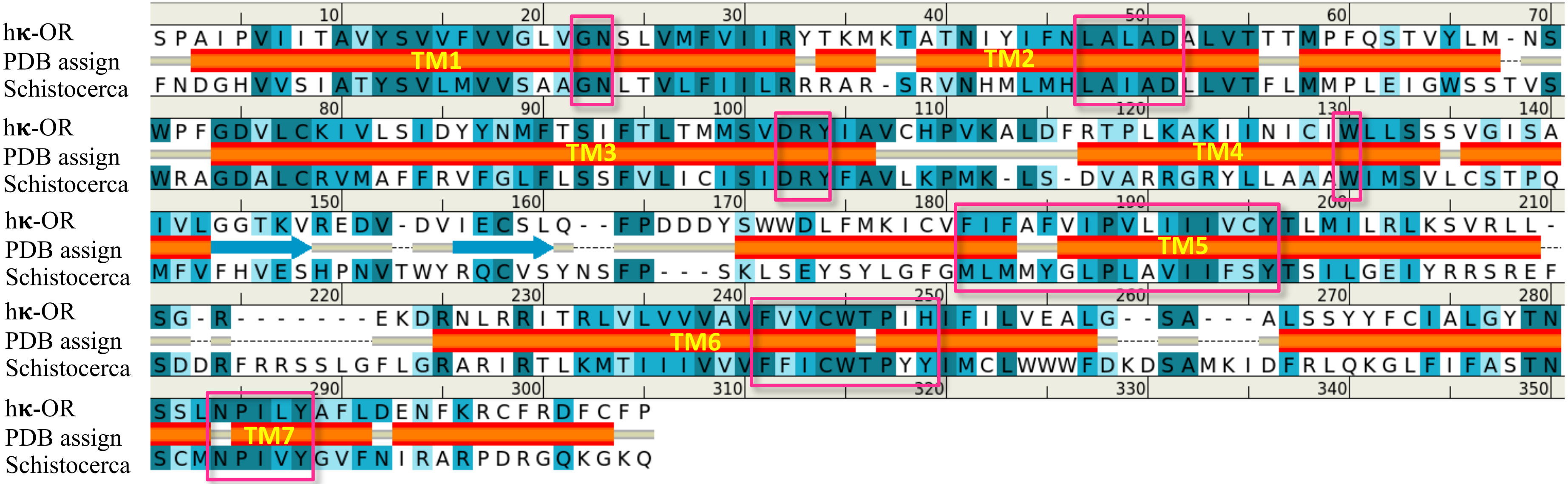

Figure S1 Sequence alignment of *Schistocerca gregaria* receptor with the crystal structures of hκ-OR (PDB ID: 4DJH). Coloring scheme indicates the degree of similarity at each alignment column. Identical (strong blue background), strongly similar (light blue background), weakly similar (very light blue background) and non-matching residues (white background) are highlighted. Experimentally determined secondary structures for hκ-OR are shown in cartoon are color coded, with helices in red, strands in blue, and coils in beige. The seven transmembrane helices (TM1-TM7) are highlighted and highly conserved residues among Class A GPCRs in hκ-OR and purple are represented in green colored boxes.

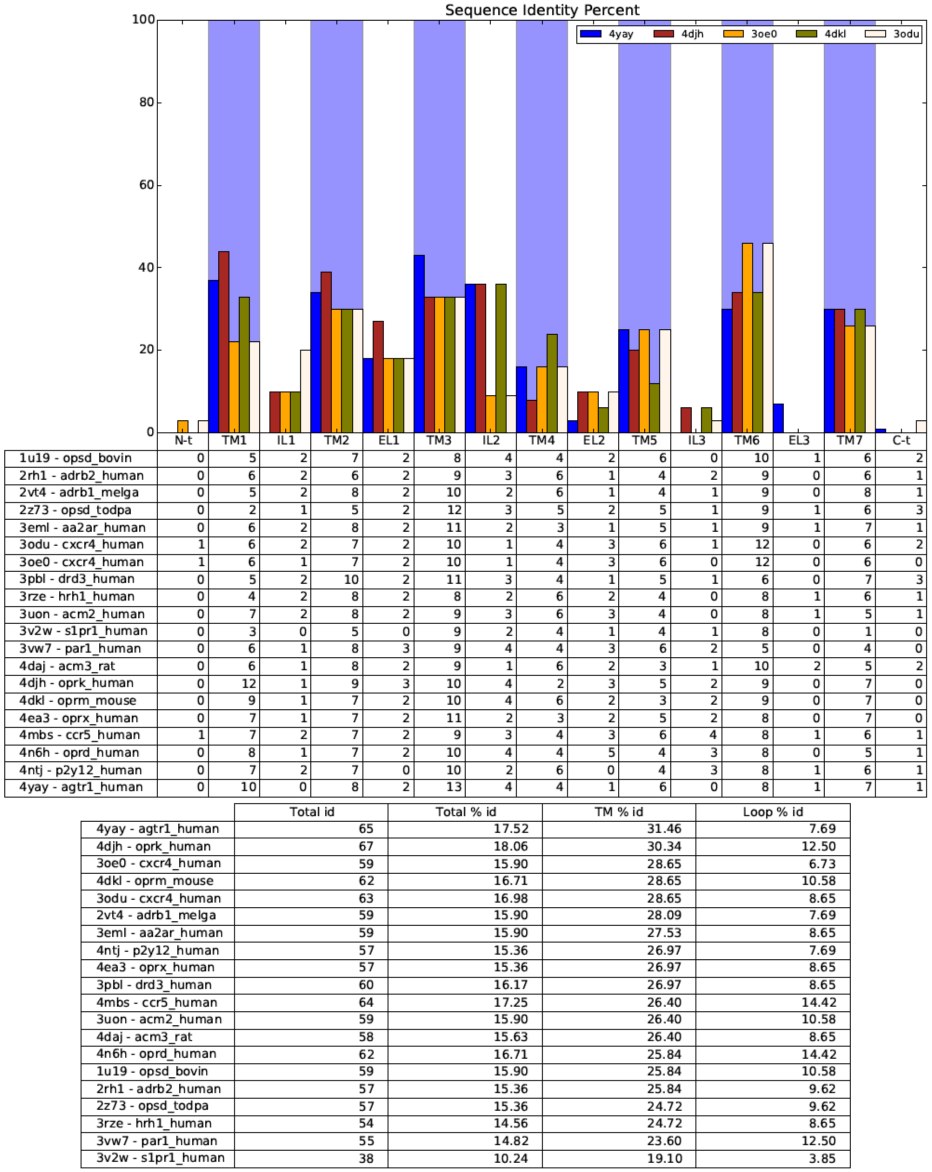

	N-t	TM1	IL1	TM2	EL1	TM3	IL2	TM4	EL2	TM5	IL3	TM6	EL3	TM7	C-t
1u19 - opsd_bovin	0	5	2	7	2	8	4	4	2	6	0	10	1	6	2
2rh1 - adrb2_human	0	6	2	6	2	9	3	6	1	4	2	9	0	6	1
2vt4 - adrb1_melga	0	5	2	8	2	10	2	6	1	4	1	9	0	8	1
2z73 - opsd_todpa	0	2	1	5	2	12	3	5	2	5	1	9	1	6	3
3eml - aa2ar_human	0	6	2	8	2	11	2	3	1	5	1	9	1	7	1
3odu - cxcr4_human	1	6	2	7	2	10	1	4	3	6	1	12	0	6	2
3oe0 - cxcr4_human	1	6	1	7	2	10	1	4	3	6	0	12	0	6	0
3pbl - drd3_human	0	5	2	10	2	11	3	4	1	5	1	6	0	7	3
3rze - hrh1_human	0	4	2	8	2	8	2	6	2	4	0	8	1	6	1
3uon - acm2_human	0	7	2	8	2	9	3	6	3	4	0	8	1	5	1
3v2w - s1pr1_human	0	3	0	5	0	9	2	4	1	4	1	8	0	1	0
3vw7 - par1_human	0	6	1	8	3	9	4	4	3	6	2	5	0	4	0
4daj - acm3_rat	0	6	1	8	2	9	1	6	2	3	1	10	2	5	2
4djh - oprk_human	0	12	1	9	3	10	4	2	3	5	2	9	0	7	0
4dkl - oprm_mouse	0	9	1	7	2	10	4	6	2	3	2	9	0	7	0
4ea3 - oprx_human	0	7	1	7	2	11	2	3	2	5	2	8	0	7	0
4mbs - ccr5_human	1	7	2	7	2	9	3	4	3	6	4	8	1	6	1
4n6h - oprd_human	0	8	1	7	2	10	4	4	5	4	3	8	0	5	1
4ntj - p2y12_human	0	7	2	7	0	10	2	6	0	4	3	8	1	6	1
4yay - agtr1_human	0	10	0	8	2	13	4	4	1	6	0	8	1	7	1

	Total id	Total % id	TM % id	Loop % id
4yay - agtr1_human	65	17.52	31.46	7.69
4djh - oprk_human	67	18.06	30.34	12.50
3oe0 - cxcr4_human	59	15.90	28.65	6.73
4dkl - oprm_mouse	62	16.71	28.65	10.58
3odu - cxcr4_human	63	16.98	28.65	8.65
2vt4 - adrb1_melga	59	15.90	28.09	7.69
3eml - aa2ar_human	59	15.90	27.53	8.65
4ntj - p2y12_human	57	15.36	26.97	7.69
4ea3 - oprx_human	57	15.36	26.97	8.65
3pbl - drd3_human	60	16.17	26.97	8.65
4mbs - ccr5_human	64	17.25	26.40	14.42
3uon - acm2_human	59	15.90	26.40	10.58
4daj - acm3_rat	58	15.63	26.40	8.65
4n6h - oprd_human	62	16.71	25.84	14.42
1u19 - opsd_bovin	59	15.90	25.84	10.58
2rh1 - adrb2_human	57	15.36	25.84	9.62
2z73 - opsd_todpa	57	15.36	24.72	9.62
3rze - hrh1_human	54	14.56	24.72	8.65
3vw7 - par1_human	55	14.82	23.60	12.50
3v2w - s1pr1_human	38	10.24	19.10	3.85

Figure S2 GPCR-ModSim server sequence identity match

MEMSAT-SVM Cartoon

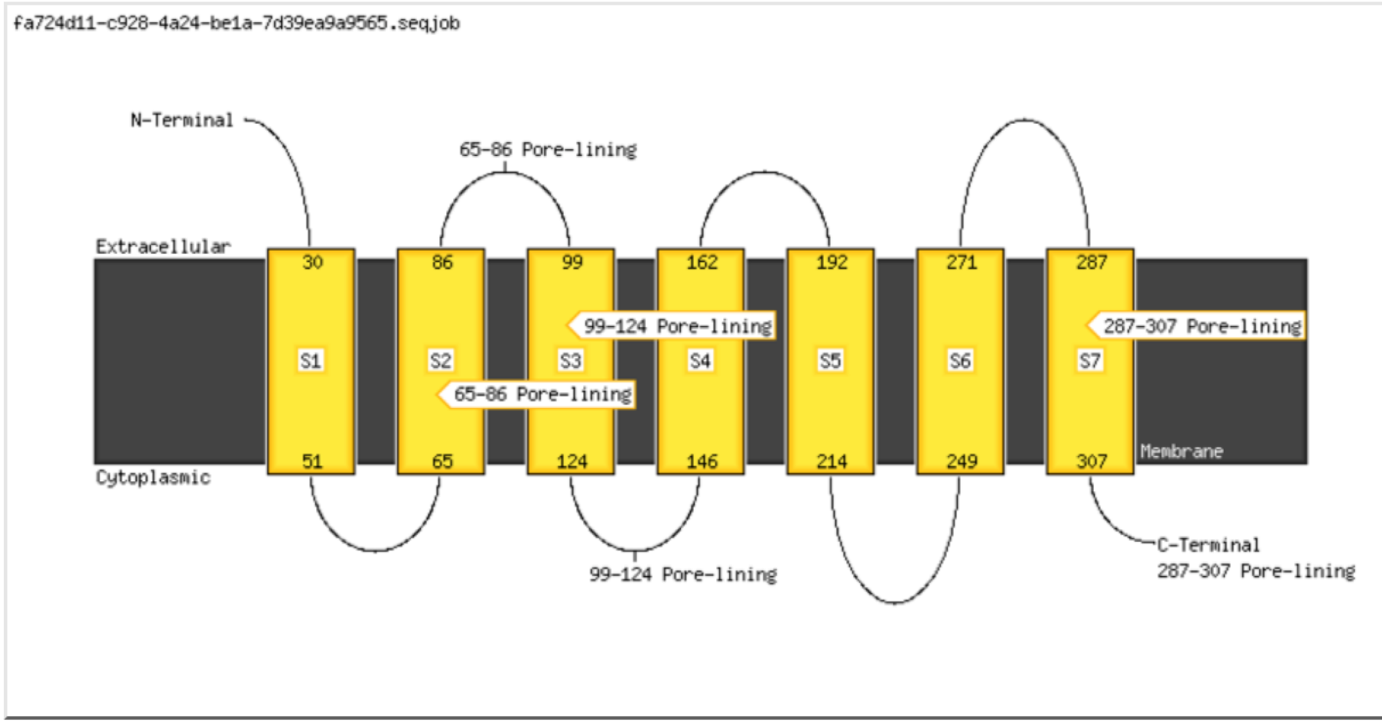

MEMSAT-SVM Prediction

Summary of MEMSAT-SVM Topology Analysis

Signal peptide	**Not detected.**
Signal score	**0**
Topology	**30-51,65-86,99-124,146-162,192-214,249-271,287-307**
Re-entrant helices	**Not detected.**
Pore-lining helices	**65-86,99-124,287-307**
Helix count	**7**
N-terminal	**out**
Score	**10.9748**
Pore stoichiometry	**1**

Figure S3. Prediction of transmembrane helices.

PROCHECK

Ramachandran Plot

8826364

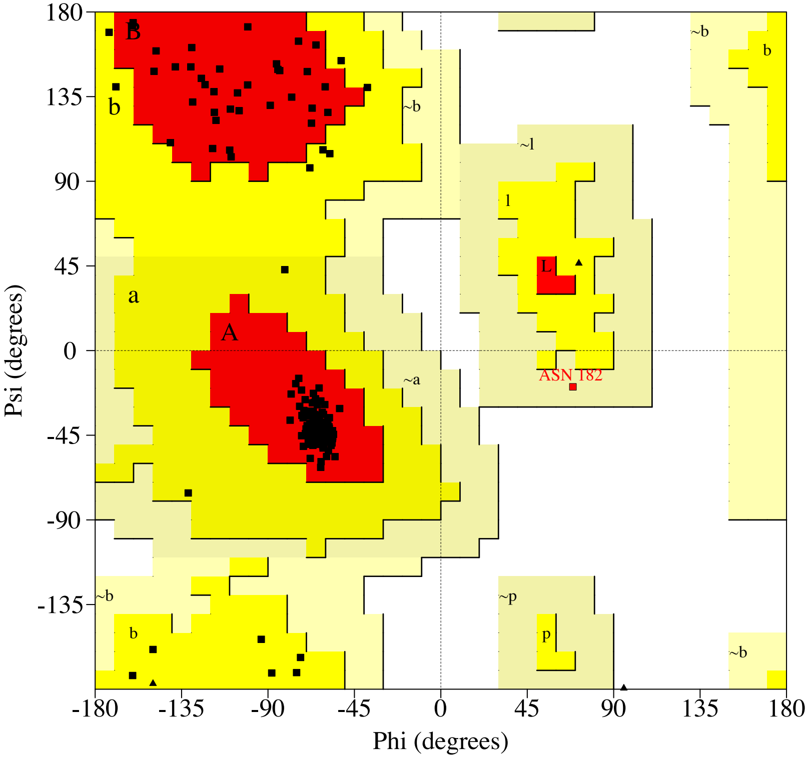

Plot statistics

Residues in most favoured regions [A,B,L]	257	94.5%
Residues in additional allowed regions [a,b,l,p]	14	5.1%
Residues in generously allowed regions [~a,~b,~l,~p]	1	0.4%
Residues in disallowed regions	0	0.0%
	----	------
Number of non-glycine and non-proline residues	272	100.0%
Number of end-residues (excl. Gly and Pro)	2	
Number of glycine residues (shown as triangles)	16	
Number of proline residues	9	

Total number of residues	299	

Based on an analysis of 118 structures of resolution of at least 2.0 Angstroms and R-factor no greater than 20%, a good quality model would be expected to have over 90% in the most favoured regions.

8826364_01.ps

Figure S4. Ramachandran plot analyses of the Schgr-AKHR model from PROCHECK

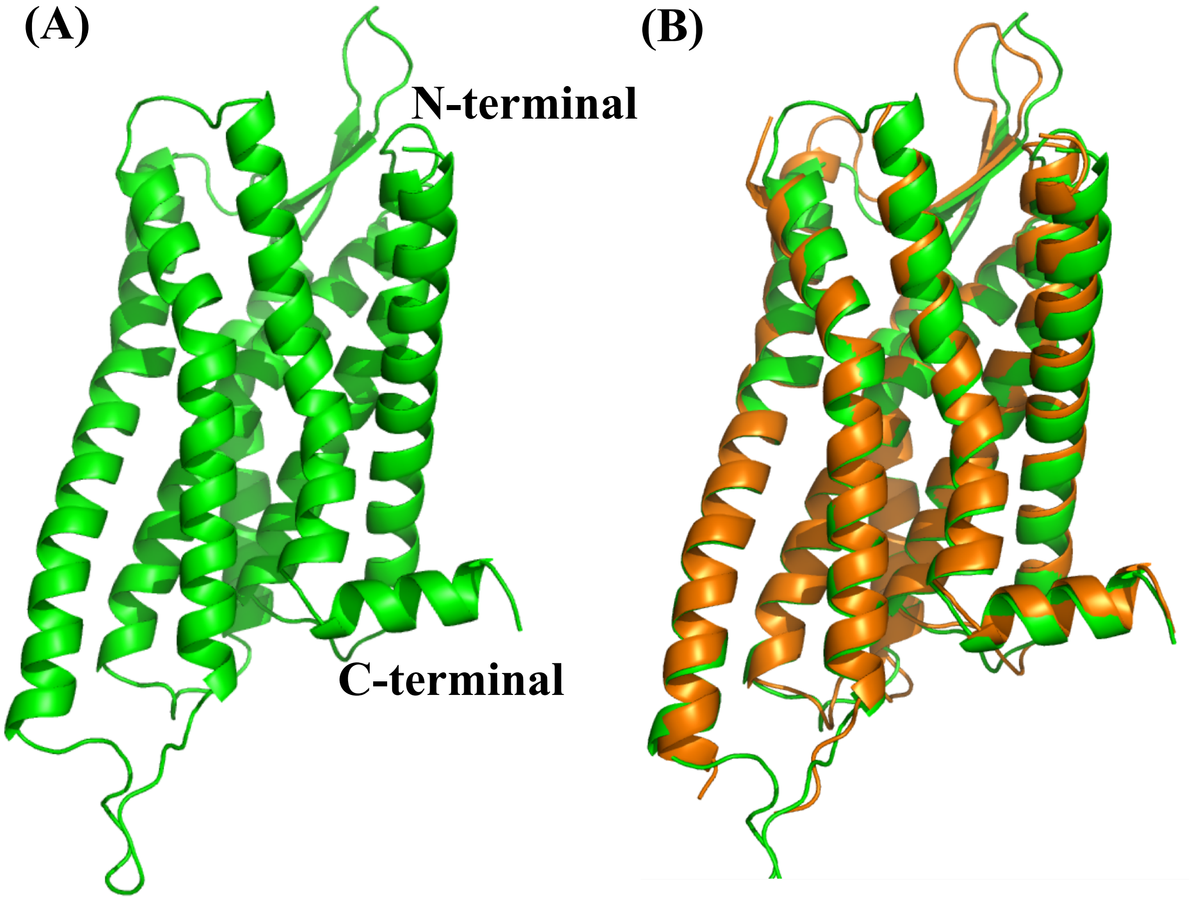

Figure S5. The final 3D structure of *Schistocerca gregaria* **(**A**)** and the comparison of *Schistocerca gregaria model* with template structure hκ-OR (orange color). Proteins are shown as ribbon diagram. The figures were made using PyMol (http://pymol.org/)
